# Gender and ethnic differences in chronic myelogenous leukemia prognosis and treatment response: a single-institution retrospective study

**DOI:** 10.1186/1756-8722-2-30

**Published:** 2009-07-24

**Authors:** Justin P Lee, Elliott Birnstein, David Masiello, Dongyun Yang, Allen S Yang

**Affiliations:** 1 Keck School of Medicine, University of Southern California, Los Angeles, California, USA; 2 Norris Comprehensive Cancer Center, USC, Los Angeles, California, USA; 3 The Jane Anne Nohl Division of Hematology and Center for the Study of Blood Diseases, USC, Los Angeles, California, USA; 4 Department of Preventive Medicine, USC/Norris Comprehensive Cancer Center, Los Angeles, California, USA

## Abstract

**Background:**

In the last decade the importance of ethnicity, socio-economic and gender differences in relation to disease incidence, diagnosis, and prognosis has been realized. Differences in these areas have become a major health policy focus in the United States. Our study was undertaken to examine the demographic and clinical features of chronic myelogenous leukemia (CML) patients presenting initially at the LAC+USC Medical Center, which serves an ethnically diverse population.

**Results:**

Patients were evenly split by gender, overwhelmingly Hispanic (60.9%), and quite young (median age 39, range 17–65) compared with previously reported CML patient populations. Previous CML studies identified significant anemia (Hgb <12 g/dl), significant thrombocytosis (platelets >450 × 10^9^/l), and significant leukocytosis (WBC >50 × 10^9^/l) as significant adverse pretreatment prognostic factors. Using these indicators, in addition to the validated Hasford and Sokal scores, patients were stratified and analyzed via gender and ethnicity. A significantly greater proportion of women presented with significant anemia (p = 0.019, Fisher's exact test) and significant thrombocytosis (p = 0.041, Fisher's exact test) compared to men, although no differences were found in risk stratification or treatment response. MCV values for women were significantly (p = 0.02, 2-sample t-test) lower than those for men, suggesting iron deficiency anemia. Focusing on ethnicity, Hispanics as a whole had significantly lower Hasford risk stratification (p = 0.046, Fisher's exact test), and significantly greater likelihood (p = 0.016, Fisher's exact test) of achieving 3-month complete haematological remission (CHR) compared with non-Hispanics at LAC+USC Medical Center, though differences in treatment outcome were no longer significant with analysis limited to patients treated with first-line imatinib.

**Conclusion:**

Female CML patients at LAC+USC Medical Center present with more significant adverse pre-treatment prognostic factors compared to men, but achieve comparable outcomes. Hispanic patients present with lower risk profile CML and achieve better treatment responses compared to non-Hispanic patients as a whole; these ethnic differences are no longer significant when statistical analysis is limited to patients given imatinib as first-line therapy. Our patients achieve response rates inferior to those of large-scale national studies. This constellation of findings has not been reported in previous studies, and is likely reflective of a unique patient population.

## Background

In the last decade the importance of ethnicity, socio-economic and gender differences in relation to disease incidence, diagnosis, and prognosis has been realized. Differences in these areas have become a major health policy focus in the United States. Several studies have addressed ethnic differences in the behavior and mortality of prostate cancer and breast cancer and have been able to demonstrate a trend toward more aggressive disease in an African-American population when compared to a Caucasian-non Hispanic population [1-6]. Studies have also shown that uninsured and poorer patients are far more likely to have advanced disease when diagnosed with cancer compared to those with private coverage [1]. Using SEER registry data, there is a known higher incidence of ALL among the Hispanic population, while there is a lower incidence of acute myelogenous leukaemia (AML) and chronic myelogenous leukaemia (CML) in the Hispanic population [7]. Additionally it has been shown that there is a higher incidence of acute promyelocytic leukaemia (APL) in the Hispanic population when compared to a non-Hispanic population [8].

CML is a clonal myeloproliferative malignancy caused by a specific chromosomal translocation involving chromosomes 9 and 22, giving rise to the "Philadelphia Chromosome," which fuses the two genes *bcr *and *abl *[9-14]. There are approximately 4500 patients diagnosed with CML every year in the United States, accounting for 15% of all new leukemias [14]. The median age of patients at presentation is 45–55 years old, with a slight male predominance [14]. The disease historically was characterized by an ordered progression from a chronic phase, to accelerated phase, to finally an acute "blastic" phase followed by death. Key breakthroughs in the treatment of this disease came about in the late 1980's early 1990's with bone marrow transplants [15,16], followed by the introduction of imatinib in the late 1990's, which has dramatically impacted the survival of patients with this disease, now approaching 80% at ten years [17-23]. It was recently shown that women with CML presented with more adverse risk factors, but had an advantage in overall survival during the interferon era [24]. Current research has begun to explore clinic-epidemiologic information on CML from developing countries, and a recent study has examined a cohort of Hispanic CML patients in Mexico City [25].

The Los Angeles County-University of Southern California (LAC+USC) Medical Center is a large publicly-funded urban medical center in the Los Angeles serving a diverse ethnic population with a high proportion of Hispanic and minority patients. In this study we evaluated the presenting and prognostic features of patients diagnosed at LAC+USC Medical Center with CML, including the calculation of the validated Hasford [26] and Sokal [27] prognostic scores. We evaluated the patients based on gender and ethnicity to evaluate the role these factors imparted upon CML presentation. We then correlated prognostic features with treatment response data, specifically the three-month complete haematological remission rate (CHR), to evaluate the predictive power of our pre-treatment analysis.

## Results

### Unique characteristics of CML patients in Los Angeles

Notable features of our overall CML patient cohort at LAC+USC Medical Center are summarized in Table 1. The median age of our CML patient group is 39 years old, with a range of 17–65 years old. Patient gender distribution is approximately equal. The patient population is overwhelmingly Hispanic in ethnicity (60.9%), reflecting the overall patient population served by LAC+USC Medical Center. Of our non-Hispanic patients, the next most common ethnicity is Asian (23.0%), followed by African American (11.5%), and lastly Caucasian (4.6%). A majority of the patients for whom treatment information is available were treated with imatinib mesylate (Gleevec) – 62.5%; virtually all patients treated with other regimens presented to LAC+USC prior to the introduction of this drug. The vast majority (91.9%) of patients presented in chronic phase CML.

**Table 1:** Overall CML Patient Characteristics (n = 87 patients)

**Characteristic**	**# of Patients (%)**
Age in years (n = 86)	
<60	79 (91.9%)
>/=60	7 (8.1%)
Gender (n = 87)	
Male	44 (50.6%)
Female	43 (49.4%)
Ethnicity (n = 87)	
Hispanic	53 (60.9%)
Non-Hispanic	34 (39.1%)
White	4 (4.6%)
African American	10 (11.5%)
Asian	20 (23.0%)
Imatinib Treatment (n = 64)	
Yes	40 (62.5%)
No	24 (37.5%)
Hemoglobin (g/dL) (n = 83)	
<12	57 (68.7%)
>/=12	26 (31.3%)
WBC count (× 10^9^/L) (n = 85)	
<50	22 (25.9%)
>/=50	63 (74.1%)
Platelets (× 10^9^/L) (n = 82)	
<450	50 (61.0%)
>/=450	32 (39.0%)
CML Phase (n = 74)	
Chronic	68 (91.9%)
Accelerated	4 (5.4%)
Blastic	2 (2.7%)
Hasford Score (n = 64)	
</=780 low-risk	35 (54.7%)
>780 and </=1480 intermediate	21 (32.8%)
>1480 high-risk	8 (12.5%)
Sokal Score (n = 65)	
<0.8 good prognosis	25 (38.5%)
0.8–1.2 moderate prognosis	24 (36.9%)
>1.2 poor prognosis	16 (24.6%)
CHR at 3 months, all treatments (n = 47)	
Yes	28 (59.6%)
No	19 (40.4%)
CHR at 3 months, imatinib only (n = 36)	
Yes	27 (75%)
No	9 (25%)

Demographic and clinical features of CML patients presenting initially at LAC+USC Medical Center are presented.

Significant proportions of our CML cohort present with hematological features identified in previous studies as significant adverse pre-treatment factors, such as a low hemoglobin, high platelet count, and high white blood cell count (Table 1). For those patients for whom we were able to calculate the validated Hasford and Sokal scores, patients trended toward the lower-risk categories by both measures. For those patients for whom we had sufficient treatment response data, the majority (59.6%) achieved complete hematologic remission (CHR) at 3 months. When analysis is limited to patients who were given imatinib as first-line treatment, overall 3 month CHR is 75%. These overall results compare unfavourably with treatment response rates achieved in large-scale national trials, which have reported three-month CHR rates of up to 86% for CML patients treated with imatinib after interferon therapy, and overall CHR rates of 95% for the entire trial duration [21,22].

### Gender-based differences in CML presentation and treatment response

Notable characteristics of our CML patient population, stratified by gender, are presented in Table 2. Differences in age and ethnic distribution are not significant. A significantly greater proportion (p = 0.019, Fisher's exact test) of females compared to males present with significantly low hemoglobin (<12 g/dL). An analysis of our MCV data found that values for women were significantly (p = 0.02, 2-sample t-test) lower than those for men, suggesting the presence of iron-deficiency anemia as a potential contributing factor. Additionally, a significantly greater proportion (p = 0.041, Fisher's exact test) of females compared to males present with significantly elevated platelet counts (>450 × 10^9^/L). Proportional differences for white blood cell count data are not significant. These three characteristics were identified in previous studies as significant adverse pre-treatment prognostic factors [27-29]. Gender-based differences between mean values for haematological data, relative Hasford and Sokal score distributions, and relative rates of 3-month CHR, stratified by imatinib treatment or overall, were not significant.

**Table 2:** Patient Stratification by Gender

	**# of Patients (%)**		
**Characteristic**	**Female**	**Male**	**P Value**
Age (yrs) (n = 86)			0.26
<60	37 (88.1%)	42 (95.5%)	
>/=60	5 (11.9%)	2 (4.5%)	
Ethnicity (n = 87)			0.19
Hispanic	23 (53.5%)	30 (68.2%)	
Non-Hispanic	20 (46.5%)	14 (31.8%)	
Hemoglobin (g/dL) (n = 83)			**0.019**
<12	34 (80.9%)	23 (56.1%)	
>/=12	8 (19.1%)	18(43.9%)	
Leukocyte count (× 10^9^/L) (n = 85)			0.46
<50	13 (30.2%)	9 (21.4%)	
>/=50	30 (69.8%)	33 (78.6%)	
Platelets (× 10^9^/L) (n = 82)			**0.041**
<450	20 (48.8%)	30 (73.2%)	
>/=450	21 (51.2%)	11 (26.8%)	
Hasford Score (n = 64)			1
</=780 low-risk	19 (52.8%)	16 (57.1%)	
>780 and </=1480 intermediate	12 (33.3%)	9 (32.1%)	
>1480 high-risk	5 (13.9%)	3 (10.7%)	
Sokal Score (n = 65)			0.39
<0.8 good prognosis	12 (33.3%)	13 (44.8%)	
0.8–1.2 moderate prognosis	16 (44.4%)	8 (27.6%)	
>1.2 poor prognosis	8 (22.2%)	8 (27.6%)	
CHR at 3 months, all treatments (n = 47)			0.23
Yes	13 (50%)	15 (71.4%)	
No	13 (50%)	6 (28.6%)	
CHR at 3 months, imatinib only (n = 36)			0.13
Yes	12 (63.2%)	15 (88.2%)	
No	7 (38.4%)	2 (11.8%)	

Selected demographic and clinical features of CML patients are presented, stratified by gender.

### Ethnicity-based differences in initial CML patient presentation

Notable characteristics of our CML patient population, stratified by ethnicity, are presented in Table 3. Differences in age and gender distribution are not significant. Ethnicity-stratified differences in mean hemoglobin, leukocyte, and platelet counts were not significant. The proportions of patients in different clinical categories for these haematological measures was also insignificant. These three characteristics were identified in previous studies as significant negative pre-treatment prognostic factors [27-29]. Ethnicity-stratified Hasford score distributions were significantly different (p = 0.046, Fisher's exact test), with Hispanic patient scores distributed more heavily in lower-risk categories compared to non-Hispanic patients. Ethnicity-stratified Sokal scores nonsignificantly trended toward the same results (p = 0.11, Fisher's exact test), with Hispanic patient scores trending towards lower-risk categories compared to non-Hispanic patients. When looking at all patients, a significantly greater proportion (p = 0.016, Fisher's exact test) of Hispanic patients achieved CHR at 3 months compared to non-Hispanic patients as a whole. When analysis is limited to patients given imatinib as first-line treatment, these differences are no longer significant (p = 0.23, Fisher's exact test)

**Table 3:** Patient Stratification by Ethnicity

	**# of Patients (%)**		
**Characteristic**	**Hispanic**	**NH**	**P Value**
Age (yrs) (n = 86)			1
<60	49 (92.5%)	30 (90.9%)	
>/=60	4 (7.5%)	3 (9.1%)	
Gender (n = 87)			0.19
Female	23 (43.4%)	20 (58.8%)	
Male	30 (56.6%)	14 (41.2%)	
Hemoglobin (g/dL) (n = 83)			1
<12	34 (68%)	23 (69.7%)	
>/=12	16 (32%)	10 (30.3%)	
Leukocyte count (× 10^9^/L) (n = 85)			0.13
<50	10 (10.6%)	12 (35.3%)	
>/=50	41 (80.4%)	22 (64.7%)	
Platelets (× 10^9^/L) (n = 82)			0.64
<450	29 (58%)	21 (65.6%)	
>/=450	21 (42%)	11 (34.4%)	
Hasford Score (n = 64)			**0.046**
</=780 low-risk	23 (56.1%)	12 (52.2%)	
>780 and </=1480 intermediate	16 (39.0%)	5 (21.7%)	
>1480 high-risk	2 (4.9%)	6 (26.1%)	
Sokal Score (n = 65)			0.11
<0.8 good prognosis	19 (45.2%)	6 (26.1%)	
0.8–1.2 moderate prognosis	16 (38.1%)	8 (34.8%)	
>1.2 poor prognosis	7 (16.7%)	9 (39.1%)	
CHR at 3 months, all treatments (n = 47)			**0.016**
Yes	20 (76.9%)	8 (38.1%)	
No	6 (23.1%)	13 (61.9%)	
CHR at 3 months, imatinib only (n = 36)			0.23
Yes	19 (82.6%)	8 (61.5%)	
No	4 (17.4%)	5 (38.5%)	

Selected demographic and clinical features of CML patients are presented, stratified by ethnicity.

### Validation of Calculated Hasford and Sokal Scores

Statistical analysis was performed to validate our calculated Hasford and Sokal scores for our CML patient cohort, as presented in Table 4. When considering all treatments, lower risk-profile Hasford scores were significantly (p = 0.035, Fisher's exact test) associated with greater likelihood of achieving 3-month CHR, while lower risk-profile Sokal scores were also significantly (p = 0.015, Fisher's exact test) associated with a greater likelihood of achieving 3-month CHR. When analysis is limited to patients given imatinib as first-line treatment, these differences remain significant for both the Hasford (p = 0.045, Fisher's exact test) and the Sokal (p = 0.0043, Fisher's exact test) scores.

**Table 4:** Validation of Calculated Hasford and Sokal Scores

	**3-mo CHR, all treatments (n = 35)**			**3-mo CHR, imatinib only (n = 32)**		
	**Yes**	**No**	**P Value**	**Yes**	**No**	**P Value**
Hasford Score			**0.035**			**0.0454**
</=780 low-risk	17 (85%)	3 (15%)		16 (88.9%)	2 (11.1%)	
>780 and </=1480 intermediate	5 (41.7%)	7 (58.3%)		5 (45.5%)	6 (55.5%)	
>1480 high-risk	2 (66.7%)	1 (33.3%)		2 (66.7%)	1 (33.3%)	
Sokal Score			**0.015**			**0.0043**
<0.8 good prognosis	14 (93.3%)	1 (6.7%)		13 (100%)	0 (0%)	
0.8–1.2 moderate prognosis	7 (58.3%)	5 (41.7%)		7 (58.3%)	5 (41.7%)	
>1.2 poor prognosis	3 (37.5%)	5 (62.5%)		3 (42.9%)	4 (57.1%)	

Achievement of 3 month CHR is presented, stratified by Hasford and Sokal risk profile categories.

## Discussion

In this current study we review several of the prognostic presentation factors of a large population of mainly Hispanic patients diagnosed and treated at LAC+USC Medical Center. An interesting observation from our study is the striking average age difference in this largely Hispanic population compared to data in the SEER registry [7], although our cohort's median age is concordant with a Hispanic CML cohort studied in Mexico City [25]. SEER registry data suggests that the incidence of CML increases with age in all ethnicities with a greater incidence in the Caucasian-non Hispanic population [7]. Additionally, our population's overall response to treatment, as measured by 3-month CHR rates, is inferior compared to those found in previous large-scale trials, which have set imatinib as the benchmark in CML therapy [17-23].

In this study we have confirmed in our largely Hispanic patient population that women present with higher risk features, specifically a higher proportion of patients relative to men, in low hemoglobin and high platelet count categories. These factors have been demonstrated in prior studies to have an adverse effect upon outcomes [27-29]. In our largely Hispanic population of patients we have shown that associations between female gender and lowered hemoglobin, as well as elevated platelet count, seen in other large CML studies [24] hold true in our population. In fact, we suspect that the main contributing factor to females presenting with lower hemoglobin and higher platelet counts is iron deficiency. In our population, iron studies were lacking in the majority of patient records, but when using mean corpuscular volume, a surrogate for iron status, we see that women present with a significantly lower MCV than compared to men. It is not clear what role, if any, iron deficiency would play in the pathogenesis and progression of CML. Despite the presence of these features, however, our study was unable to demonstrate gender-stratified differences in Hasford or Sokal score distribution, or in treatment outcome as defined by 3-month CHR. This is at odds with previous studies [24], which found less favourable risk profiles, but longer survival times, during the interferon era. We postulate that these differences may be attributable to the unique ethnic and socio-economic profile of our patient population, as well the advent of imatinib, a highly efficacious treatment not yet developed at the time that the Hasford and Sokal scores were validated.

We demonstrated lower risk stratification scores for Hispanic patients versus non-Hispanic patients combined. Hispanic patients were also more likely to achieve 3 month CHR compared to non-Hispanic patients when all patients were analysed, although these differences were no longer significant when analysis was limited to patients given imatinib as first-line treatment. These differences may more likely be due to non-Hispanic ethnicities presenting and performing more poorly than the norm, rather being attributable to superior prognostic features and outcomes for Hispanic patients. The vast majority of our non-Hispanic patients were African American or Asian. Indeed, our results may reflect a trend of non-Hispanic minority CML patients presenting and performing more poorly than national averages. We believe that socio-economic factors may play a role in explaining these differences. Additional studies are ongoing. Additionally, the disparities between risk stratification and treatment outcome in imatinib-treated patients may be attributed to the advent of imatinib as first-line therapy for CML, not yet developed at the time that the Hasford and Sokal scores were validated. However, we cannot rule out the possibility that perhaps a biological/genetic factor also contributes to this observed ethnic differences in disease presentation and behaviour, as shown previously at our institution for APL in the Hispanic patient populations [8].

Our cohort as a whole, achieved 3-month CHR rates inferior to those examined in previous large-scale CML trials, which have yielded 3-month CHR rates of up to 86 percent [17-23]. To our knowledge this is the first report of a large cohort of Hispanic patients with CML diagnosed in the United States. There has been a recent study examining a CML cohort at a single institution in Mexico City [25]. Additionally, there are several studies addressing Hispanic patients and transplant outcome in leukemia in general with a small sub population of patients with CML [30-33]. These studies, however, suggest that Hispanic patients have a worse outcome following transplantation and may have more barriers to this treatment option when compared to Caucasian non-Hispanic patients. In these studies presenting factors were not addressed. The variation between these studies and our own findings may be attributable to the factors discussed above, and may simply be findings unique to our patient population. Additional studies are ongoing.

We acknowledge that our study is limited by numerous logistical difficulties. Complete data sets were not available for all 87 patients identified in this study, limiting the number of patients for whom we could provide a full demographic profile and calculate Sokal and Hasford scores. Spleen sizes were not available for the vast majority of patients, so an average value was interpolated for the purpose of calculating Sokal and Hasford scores. Additionally, treatment response and long-term followup data was not available for numerous patients included in the pre-treatment analysis, limiting our statistical power in analysing the association between pre-treatment prognostic factors and treatment response. We were, however, able to validate our Hasford, Sokal, and treatment response data, as lower risk profiles were appropriately significantly associated with better treatment response, both when looking at all patients regardless of treatment, and when analysis was limited to patients given imatinib as first-line treatment.

## Conclusion

We conclude that female CML patients at LAC+USC Medical Center present with more significant adverse pre-treatment prognostic factors identified in previous studies compared to men, while Hispanic patients present with lower risk profile CML and achieve better treatment responses compared to our non-Hispanic patients as a whole; these ethnic differences are no longer significant when statistical analysis is limited to patients given imatinib as first-line therapy. Our cohort as a whole failed to achieve response rates seen in large-scale national studies [17-23]. This constellation of findings has not been reported in previous studies, and is likely reflective of a unique patient population.

We found that women were more likely to present with significant anemia and significant thrombocytosis. An analysis of our MCV data found that values for women were significantly (p = 0.02, 2-sample t-test) lower than those for men, suggesting that the gender differences observed are likely due to iron deficiency anemia, with reactive thrombocytosis. At the same time, however, there were no significant gender-based differences in Hasford and Sokal risk stratification, or in treatment response. To our knowledge, this combination of findings has not been reported in previous studies.

Surprisingly, we also found that Hispanic CML patients had lower risk stratification scores and correspondingly greater likelihood of achieving 3-month CHR compared to non-Hispanic patients as a whole when all patients are analysed regardless of treatment type; these ethnic differences in treatment response are no longer significant when analysis is limited to patients treated with imatinib as first-lined therapy. These are novel findings that have not been reported in previous studies, and may reflect a trend of our non-Hispanic minority patients presenting and performing more poorly than national norms. Although true biological differences may play a role, we believe that such differences may be attributable to the unique ethnic and socio-economic profile of our patient population at LAC+USC Medical Center, underscoring the need for further study into the significance of these factors.

## Methods

### Collection and Analysis of Pre-treatment Data

We began by establishing a database of all patients presenting with CML at LAC+USC Medical Center between the years 1991–2008. We identified and obtained adequate medical records for 87 CML patients. Data collected included gender, ethnicity, city and zip code of residence, date of birth, date of diagnosis, treatment, course of treatment, and any noted side effects, as well as complete blood count data at presentation (pre-treatment), comprised of hemoglobin (Hgb), hematocrit, mean corpuscular volume (MCV), mean corpuscular hemoglobin, mean corpuscular hemoglobin concentration, platelet count, white blood cell count (WBC), and a WBC differential. Hasford and Sokal scores were calculated for all patients as available data allowed. As spleen size was not reliably or routinely recorded in our medical records, the mean spleen size reported in the original Hasford study [26] was used as a proxy in calculating the Hasford score of all patients.

This data was then analysed in an overall context, as well as by gender and ethnicity based stratifications, to yield our pre-treatment findings for this study. Patients who were previously diagnosed with CML at an outside institution and undergoing treatment at time of presentation to LAC+USC Medical Center were excluded from pre-treatment prognostic analysis. The associations between patient baseline demographic and clinical characteristics, and gender and ethnicity were examined using contingency tables and Fisher's exact tests. The MCV values were compared using a 2-sample *t *test.

### Collection and Analysis of Treatment Response Data

Treatment response was determined by assessing complete hematologic response (CHR) after three months of treatment. CHR was defined as a complete blood count (CBC) with a white blood cell count below 10 × 10^9^/L and a differential of 0% immature granulocytes, a platelet count below 450 × 10^9^/L and less than 5% basophils with a non-palpable spleen [23]. A chart review was performed for each of the 87 CML patients in the database and three month complete blood counts (CBC) were collected to assess CHR status, although complete CBC data were available for only 47 of the 87 patients. Spleen palpability for individual patients could not be assessed due to limitations discussed previously and was not included as a variable in the final analysis. Data was collected from both LAC-USC's computer-based charting system as well as hand-written charts that were recorded before the computer system was in place.

A contingency table and Fisher's exact were used to evaluate the associations of prognostic factors, sex, and ethnicity with CHR.

The 5% level of significance was used and *p*-values were 2-sided. All analyses were performed using the SAS statistical package version 9.1 (SAS Institute Inc. Cary, North Carolina, USA).

### IRB Approval

This study was conducted with Institutional Review Board approval under protocol HS-06-00258, "Ethnic Differences in Hematologic Malignancies."

## Competing interests

The authors declare that they have no competing interests.

## Authors' contributions

JPL collected the pre-treatment data used in the study, compiled the CML patient database, participated in the study design, and drafted the manuscript. EB collected the treatment response data used in the study, and helped draft the manuscript. DM undertook background research for the study, assisted in the statement of findings, and helped to draft the manuscript. DY performed the statistical analyses utilized in the study, and helped to draft the manuscript. ASY conceived of the study, participated in its design and coordination and helped to draft the manuscript. All authors read and approved the final manuscript.
